# Study on Microscopic Oil Displacement Mechanism of Alkaline–Surfactant–Polymer Ternary Flooding

**DOI:** 10.3390/ma17184457

**Published:** 2024-09-11

**Authors:** Guoqiao Li, Zhaohui Zhou, Jian Fan, Fan Zhang, Jinyi Zhao, Zhiqiu Zhang, Wei Ding, Lu Zhang, Lei Zhang

**Affiliations:** 1 College of Chemistry and Chemical Engineering, Northeast Petroleum University, Daqing 163318, China; liguoqiao_2001@126.com (G.L.); zhang_zhiqiu@126.com (Z.Z.); 2 No. 2 Oil Production Plant, Daqing Oilfield Corp., Ltd., Daqing 163414, China; 18904893123@189.cn; 3 State Key Laboratory of Enhanced Oil & Gas Recovery, PetroChina Research Institute of Petroleum Exploration & Development, Beijing 100083, China; zhouzhaohui@petrochina.com.cn (Z.Z.); fanjian@petrochina.com.cn (J.F.); zhangfan902@petrochina.com.cn (F.Z.); 4 Key Laboratory of Photochemical Conversion and Optoelectronic Materials, Technical Institute of Physics and Chemistry, Chinese Academy of Sciences, Beijing 100190, China; luyiqiao@mail.ipc.ac.cn

**Keywords:** alkylbenzene sulfonate, weak alkaline, ASP flooding, ternary system, emulsification, oil displacement

## Abstract

Alkali–surfactant–polymer (ASP) flooding is one of the most effective and promising ways to enhance oil recovery (EOR). The synergistic effect between alkali, surfactant, and polymer can respectively promote emulsification performance, reduce interfacial tension, and improve bulk phase viscosity, thus effectively improving flooding efficiency. However, the displacement mechanism of ASP flooding and the contribution of different components to the oil displacement effect still need further discussion. In this study, five groups of chemical slugs were injected into the fracture model after water flooding to characterize the displacement effect of weak alkali, surfactant, polymer, and their binary/ternary combinations on residual oil. Additionally, the dominant mechanism of the ASP flooding system to improve the recovery was studied. The results showed that EOR can be improved through interfacial reaction, low oil/water interfacial tension (IFT), and increased viscosity. In particular, the synergistic effect of ASP includes sweep and oil washing. As for sweep, the swept volume is expanded by the interfacial reaction between the alkali and the acidic components in Daqing crude oil, and the polymer increases the viscosity of the system. As for oil washing, the surfactant generated by the alkali cooperates with surfactants to reduce the IFT to an ultra-low level, which promotes the formation and migration of oil-in-water emulsions and increases the efficiency of oil washing. Overall, ASP can not only activate discontinuous oil ganglia in the pores within the water flooding range, but also emulsify, decompose, and migrate the continuous residual oil in the expanded range outside the water flooding. The EOR of ASP is 38.0% higher than that of water flooding. Therefore, the ASP system is a new ternary composite flooding technology with low cost, technical feasibility, and broad application prospects.

## 1. Introduction

Crude oil is an important energy resource related to the sustainable development of the world. As production demand increases, output is gradually decreasing [1]. Chemical flooding after water flooding is one of the most effective ways to enhance oil recovery (EOR), including alkali flooding, polymer flooding, surfactant flooding, binary (surfactant–polymer) flooding, and ternary (alkali–surfactant–polymer) flooding [2,3]. For conventional surfactant flooding, the displacement effect is greatly affected by the concentration window, which may result in an unsatisfactory oil displacement effect at low concentrations. Comparatively, high concentrations not only directly increase economic costs but are also limited by the influence of the water/oil mobility ratio [4]. To solve the above problems, it is necessary to add surfactants or polymers to increase the viscosity of the system.

For alkali flooding, alkali can react with the acidic components in crude oil to generate active substances in situ, reducing the interfacial tension (IFT) of crude oil to an ultra-low level (10^−3^ mN/m). In addition, active substances can increase the capillary number, reduce the mobilization energy of crude oil, promote the activation of residual oil retained in the reservoir pores, and significantly reduce the residual oil saturation [5,6]. However, strong alkalis will cause scaling, corrosion, and clay swelling, resulting in reduced permeability and damage to the formation [7,8]. For polymer flooding, the elastic effect increases the flow resistance, reduces the mobility ratio, and blocks the large pores through adsorption and retention, thereby improving the sweep effect [9,10]. Torrealba et al. [11] and Zhang et al. [12] studied the microscopic displacement mechanism of viscoelastic fluids, and they found that partially hydrolyzed and modified polymers can produce higher pressure drop through viscoelastic effects, change the flow direction, and increase the swept volume. In addition, the strong shear stress generated by porous media can also pull the residual oil into small oil ganglia to mobilize the remaining oil. The core mechanism of polymer flooding is to improve the displacement front morphology of the injected fluid, which plays a role in dragging and stripping during the migration process. However, the disadvantage is that it is almost impossible to mobilize isolated oil ganglia [13]. In other words, polymer flooding has a lower displacement efficiency in small pores where the capillary force is dominant over the viscous force. Therefore, surfactants or alkalis can be added to polymers to reduce IFT, promote emulsification performance, and improve interfacial properties such as bulk viscosity. Thus, the oil displacement performance limitations of alkali (A), surfactants (S), and polymers (P) are supplemented and improved [14,15]. Fu et al. studied the microscopic oil displacement mechanism of the ethanolamine–petroleum sulfonate–partially hydrolyzed polyacrylamide (OASP) system through microscopic displacement and sandpack experiments. It was found that the petroleum sulfonate and alkali synergistically produced ultra-low IFT, emulsified crude oil into W/O emulsion to increase the flow resistance of the fluid, and assisted the polymer to improve mobility and thus expanded the swept volume. As a result, the oil recovery was increased by 21.7% [16]. However, this also means that the oil displacement mechanism of the ASP system and the contribution of each component to the mobilization of residual oil in water flooding are complicated [17,18].

Numerous studies have shown that ASP flooding can effectively improve the recovery rate of Daqing Oilfield [19,20,21]. ASP flooding has shifted from a strong alkaline type to a weak alkaline type (Na_2_CO_3_) [22]. In the ternary system, there is a synergistic effect between the alkali, surfactant, and polymer, which can effectively improve oil recovery. Alkali can react with organic acids in crude oil to form surfactants in situ. The synergies between alkali and surfactants produce ultra-low interfacial tension. Alkali can also promote the emulsification of crude oil, change the wettability of rocks, and regulate pH and salinity. Surfactants can reduce the interfacial tension of oil and water and change the wettability of rock. The polymer can improve the viscosity of the water phase, reduce the flow of water and oil, and effectively improve the sweep efficiency. This has been confirmed in indoor exploratory research and has achieved industrial promotion [23,24,25]. Li et al. [26] and Cheng et al. [27] confirmed that the alkali–domestic alkylbenzene sulfonate–polymer ternary composite flooding system achieved an oil recovery of more than 18% higher than that of water flooding. ASP systems can simultaneously have excellent interfacial properties such as excellent IFT reduction ability, viscoelastic properties, and good emulsifying properties. However, the roles of different components in the composite flooding system are ignored to a certain extent [28]. In addition, the comparison of displacement characteristics and migration process between water flooding and composite systems also lacks systematic research, which limits the application of ASP systems. Therefore, to clarify the mechanism of improving the recovery of Daqing crude oil by the high-efficiency ternary composite system, it is necessary to study the change law of the remaining oil in the pore structure after composite flooding.

In this work, weak alkali with little damage to the formation, heavy alkylbenzene sulfonate with low cost, and hydrolyzed polymer were utilized to construct a new ternary composite flooding formula to investigate the residual oil in the pores before and after composite flooding. Based on the interfacial properties of different systems, the dynamic changes and oil displacement efficiency of the microscopic residual oil after water flooding and different chemical slug flooding were analyzed to explore the microscopic oil displacement mechanism of the ASP system for effectively mobilizing residual oil. Herein, a novel ternary composite ASP flooding formula is constructed and the microscopic oil displacement mechanism is also explored, which is of great significance for the application of the ASP flooding system in Oilfield.

## 2. Materials and Methods

### 2.1. Materials

**Figure 1:**
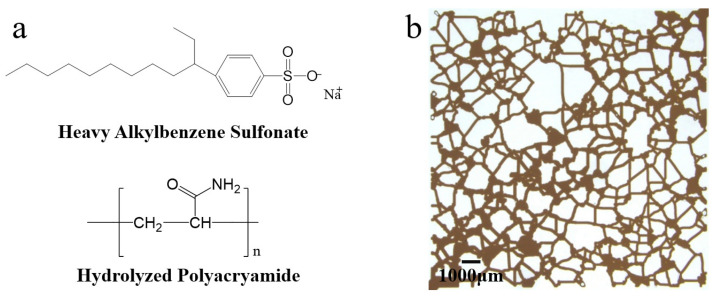
The typical chemical structures of heavy alkylbenzene sulfonate and hydrolyzed polyacrylamide (**a**), and the shape and structure of the microfluidic chip model after the saturated oil phase (**b**).

Heavy alkylbenzene sulfonate used in this study was produced by Daqing Donghao Investment Co., Ltd. (Daqing, China), and its effective component was 50%. The dehydrated and degassed crude oil with a viscosity of 57.30 cP at 25 °C and a viscosity of 20.60 cP at reservoir temperature (45 °C) in the production wells of Daqing Oilfield in China was used as the oil phase. The polymer (hydrolyzed polyacrylamide) was produced by Daqing Refining and Chemical Company, and the specific physical and chemical indicators are shown in Table 1. Na_2_CO_3_ (AR) was obtained from Modern Oriental (Beijing) Technology Development Co., Ltd. (Beijing, China). The chemical structures of heavy alkylbenzene sulfonate and hydrolyzed polyacrylamide (HPAM) are depicted in Figure 1a. The simulated formation water of the corresponding components was prepared according to the composition of the reservoir formation brine as shown in Table 2. All the displacement solutions in the ASP system were prepared using the simulated formation water. The chip model used in the visualization oil displacement experiments was provided by the China University of Petroleum (Beijing, China). The model (Figure 1b) was the water-wet type with a side length of 15.00 × 15.00 mm, pore depth of 0.03 mm, pore area of 52.53 mm^2^, and pore volume of 1.58 mm^3^.

### 2.2. Apparatus and Method

#### 2.2.1. Interfacial Tension Test

A spinning drop interfacial tensiometer (TX-500C) (Shengwei Industrial Technology Co., Ltd., Beijing, China) was used to determine the dynamic interfacial tension between oil displacement solutions and crude oil [29]. The rotation speed was 5000 r/min, and the temperature was 25 °C. The inner phase was crude oil and the outer phase was the oil displacement agent solution [30].

#### 2.2.2. Bulk Viscosity Test

The bulk viscosity tests of alkali, surfactant, polymer, SP binary, and ASP ternary flooding solutions were performed by a rotary rheometer (HAAKE MARS II) (Thermo Fisher, Karlsruhe, German) at 25 °C. The rotor type was C60/1° Ti and the speed was fixed at 7.34 1/s [31].

#### 2.2.3. Emulsifying Property

The emulsification performance and emulsion stability of different chemical slug solutions were characterized by the bottle test method, and the morphology of the emulsion was photographed [32]. First, the crude oil and different displacement solutions were added to the emulsion scale glass tube at a volume ratio of 5:5. Then, 100 oscillations were performed to ensure complete emulsification. Under the condition of 25 °C, the water distribution of the oil/water emulsion in 0~2 h was recorded, and the water separation rate was used as the evaluation standard of the solution’s emulsification performance.

#### 2.2.4. Microscopic Visualization Oil Displacement Experiment

The oil displacement experiments were completed in the chip model of the microscopic visual oil displacement system (VMF100, Eastern-Dataphy Instruments, Beijing, China) at 25 °C [1]. First, the crude oil was completely saturated in the model. Then, a microfluidic injection pump (FLOW-EZ S/N: 12,490) was used to inject the displacement fluid from the upper right inlet at a constant injection rate of 0.1 μL/min and displace from the lower left outlet. In particular, the water flooding continued to inject 1 PV, and the single chemical slug solution continued to inject 2 PV. The displacement process was recorded by a microscope (Nikon SMZ18, Nikon Corporation, Tokyo, Japan). Before the experiment, the mold chip was cleaned with petroleum ether, ethanol, and distilled water, and finally dried on a heating plate. In the experiment, the models were placed in a horizontal state.

In this study, a total of 5 groups of tests were carried out. First, the initial water flooding was carried out, and then the chemical solution slug was injected. Chemical slugs include Na_2_CO_3_ (A), heavy alkylbenzene sulfonate (S), polymer (P), and their combinations, namely SP (heavy alkylbenzene sulfonate + polymer) and ASP (Na_2_CO_3_ + heavy alkylbenzene sulfonate + polymer).

## 3. Results

### 3.1. Oil/Water Interfacial Tensions

**Figure 2:**
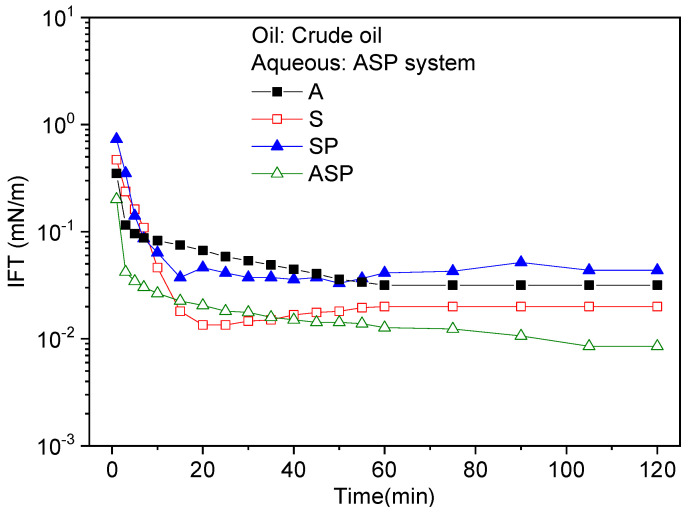
Dynamic IFTs between A/S/SP/ASP solutions and Daqing crude oil.

The IFTs between A/S/SP/ASP solutions and Daqing crude oil were measured and the results are shown in Figure 2. It can be seen that the IFT decreases with time and then reaches a plateau value, and the curve shows a typical “L” shape. The A/S/SP system can reduce the IFTs of crude oil to 10^−2^ mN/m order of magnitude, while the ASP system can quickly reduce the IFTs to an ultra-low level. The reduction of IFT can be attributed to the increase in surfactant molecules adsorbed at the oil/water interface and the compacted adsorption film [33]. In the Na_2_CO_3_ (A) system, Na_2_CO_3_ reacts in situ with petroleum acids or cyclohexane acids in crude oil to generate active substances. These substances can significantly reduce IFT by adsorbing at the oil/water interface [5]. In the alkylbenzene sulfonate (S) system, the IFT gradually decreases until it reaches the equilibrium value as the adsorption of surface molecules at the interface increases. The addition of polymers to the SP system has a significant impact on the IFT between the surfactant solution and crude oil. The main reason is that the polymer chains without interfacial activity penetrate the surfactant adsorption layer to form a mixed adsorption layer. Also, the polymer will destroy the hydrophilic–lipophilic balance and result in a loose interfacial adsorption film. As a consequence, the IFT of the composite system is higher than that of the S system. For the ASP system, first of all, the surface active substances generated by the alkali have a synergistic effect with the surfactant molecules at the interface, making the interfacial film tighter. Secondly, the polymer can protect the surfactant from reacting with Ca^2+^ and Mg^2+^. In addition, the surfactant and polymer adsorb at the oil/water interface to form a mixed adsorption layer. Generally, partially hydrolyzed polyacrylamide has multiple ion heads; accordingly, the mixed adsorption film has a higher surface charge [34]. Compared with other systems, the IFT in the ASP system is further reduced. However, in general, all systems have good interfacial activity.

### 3.2. Bulk Viscosity

**Figure 3:**
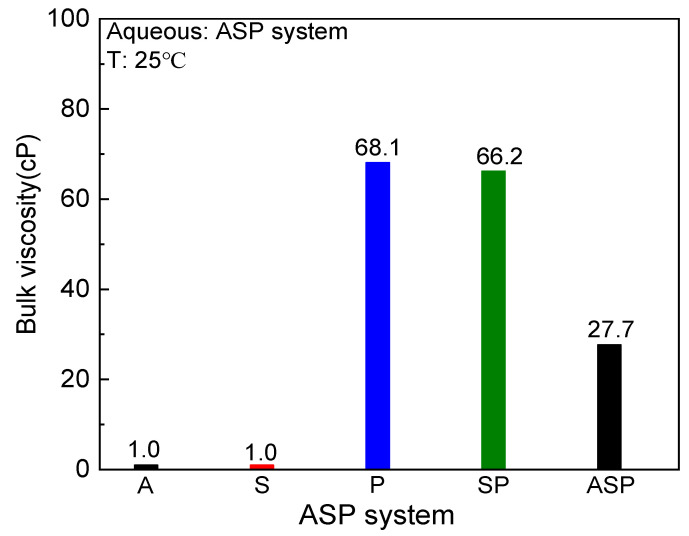
Bulk viscosity of the A/S/P/SP/ASP systems.

Bulk viscosity is also a key factor affecting the oil recovery of the ASP system, which is directly related to the flow state between the displacing phase and the displaced phase. Figure 3 shows the viscosity values of the A/S/P/SP/ASP systems at 25 °C. In Figure 3, the viscosity of the A and S systems is similar to that of distilled water, and the viscosity of the P system is the highest (68.1 cP). In comparison, the addition of surfactants slightly reduces the viscosity of the SP system (66.2 cP), but the overall difference is small. Moreover, the introduction of weak alkalis significantly reduces the viscosity of the ASP system (27.7 cP). Obviously, there are large differences in viscosity between different systems. Partially hydrolyzed polymer (HPAM) is a commonly used polymer in EOR. On the one hand, the high molecular weight of HPAM enhances its thickening ability. On the other hand, electrostatic repulsion between the same coil or polymer coils gradually stretches the polymer chain and leads to an increase in viscosity [35]. In the SP system, the surfactant interacts with the polymer chain, which affects the rheological behavior of the polymer. The heavy alkylbenzene sulfonate used in this study contains a certain amount of salt, and it is also an ionic surfactant. When the concentration is 0.3%, the heavy alkylbenzene sulfonate plays the role of salt to reduce the viscosity of the system. Xia et al. [36] studied the rheological properties between sodium oleate and HPAM, and it was confirmed that high concentrations of surfactants would increase the micelle aggregates on the HPAM chain, resulting in the shrinkage of the HPAM coil. Thus, the network between the surfactant and the polymer collapses and the viscosity decreases. The viscosity data in Figure 3 shows that the effect of alkali on the polymer in the ASP system is complex. On the one hand, the alkali plays the role of salt. On the other hand, the chain of polymers is easy to break under alkaline conditions, which leads the viscosity of the ASP system to show a significant decrease compared with the P and SP systems. Li et al. [21] also confirmed that the cations introduced by high concentrations of alkaline solutions can produce charge shielding, leading to a decrease in polymer viscosity. Moreover, the alkali promotes the hydrolysis of the amide group to relax the polymer chain, resulting in a decrease in viscosity.

### 3.3. Emulsification Performance and Emulsion Stability Test

**Figure 4:**
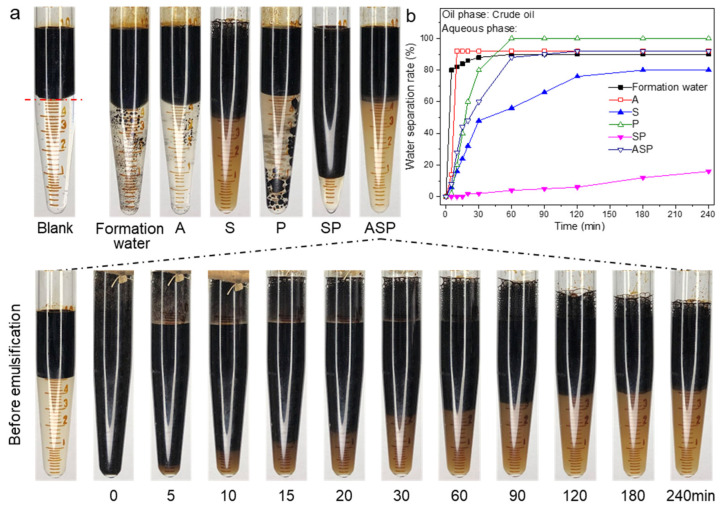
Emulsification performance (**a**) and water separation rate (**b**) of emulsions formed by the A/S/P/SP/ASP systems and crude oil. Blank represents the initial state of the water phase and oil phase.

The emulsification ability and emulsion stability also play an important role in oil recovery performance. The stability and water separation rates of emulsions formed by the formation water and the A/S/P/SP/ASP systems with crude oil are respectively shown in Figure 4a,b. In Figure 4a, formation water and crude oil will be quickly separated after shaking. It can also be seen that the final state of the water separation is close to the state before shaking (Figure 4b). Although the A solution can emulsify the crude oil, the phase separation speed is relatively fast, and its state after shaking is similar to the formation water. The S solution has a moderate degree of emulsification ability, but the emulsion lacks stability. Likewise, the P solution cannot emulsify crude oil. Although the water separation rate for the P solution slows down due to the increase in viscosity, the final state of the emulsion is still similar to the formation water. Among the six emulsification experiments, the emulsion of the SP system possesses the highest stability. This can be attributed to the emulsification ability of S and the enhanced viscosity of P, thereby the stability of the emulsion is improved [37,38]. Furthermore, the ASP system is based on the SP system with the addition of alkali. The low IFT makes the crude oil easier to emulsify, and the viscosity of the system is simultaneously reduced (Figure 3) [39,40]. As an electrolyte, alkali weakens the stability of the emulsion. However, it is visible in Figure 4a that the ASP system exhibits strong emulsification ability, fast emulsification rate, and uniform emulsion ability in the initial emulsification stage. The ASP system also has the ability to emulsify and strip crude oil, which can provide a guarantee for excellent oil recovery performance.

### 3.4. Oil Displacement Effect

For each set of chemical solution plug tests, the state of the remaining oil, the oil recovery of formation water, and a single chemical plug were analyzed and calculated. Table 3 gives the EOR results of the five tests. Due to the large difference in the water/oil mobility ratio, water flooding presents a typical fingering feature. As a result, water flooding cannot mobilize a large area of crude oil, and the average oil recovery is only about 40.6%. Specifically, after the injection of the A segment plug, the low IFT generated by the interfacial reaction can increase the oil displacement efficiency by 6.3% based on water flooding. In contrast, the displacement effect of S is not ideal even though S possesses low IFT. This is because no interfacial reaction occurs, and the shear disturbance ability of the solution is also weak. The fluid migrates along the dominant path and almost no residual oil is activated (EOR is only 0.9%). After the injection of the P solution, although its interfacial activity is poor, it can significantly improve the oil recovery by increasing the bulk viscosity of the displacement fluid, slowing down the flow rate, and increasing the sweep. Compared with A and S flooding, P flooding has a significant improvement in EOR (EOR is 13.2%) [11]. The SP system reduces the IFT value to a lower level and has a suitable viscosity. Therefore, SP flooding has a higher oil recovery than A, S, and P flooding, and SP flooding can increase the EOR to 20.9%. Furthermore, the oil recovery of the ASP combination was tested, and it was found that the 1.2% A + 0.3% S + 0.2% P chemical slug produced the highest EOR (38.0%).

In order to elucidate the oil displacement mechanism of different systems in detail, the sweep efficiency, the oil displacement efficiency within the swept range, the contribution of different chemical slugs in the water flooding range, and the expanded sweep area outside the water flooding range are respectively investigated to explore the key factors affecting the oil recovery of the ASP system.

**Figure 5:**
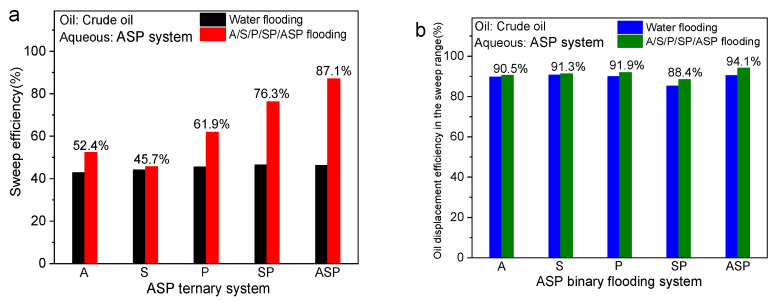
The sweep efficiency (**a**) and the oil displacement efficiency within the sweep range (**b**) calculated by the final oil displacement effects of the A/S/P/SP/ASP systems.

Based on the displacement effect in the final stage, the sweep efficiency and oil displacement efficiency within the swept range after formation water and five groups of chemical slug solutions undergo flooding are shown in Figure 5. In general, except for the S system, all systems can expand the swept volume to varying degrees based on water flooding, and the overall oil displacement efficiency within the swept range of all systems is also slightly increased. The ASP system has the best sweep effect and remaining oil capacity within the initial sweep range, as shown in Figure 5a,b.

From the sweep efficiency in Figure 5a, the remaining oil is mainly in columnar, porous, and continuous clusters, and the state of the remaining oil no longer changes after the formation water forms a fingering path. The sweep efficiency of the five groups of water flooding is between 44.1% and 46.7%, and the sweep capacity is limited. Due to the large difference between the dominant channel and the water/oil mobility ratio of the S solution, the contact between the solution and the crude oil was insufficient, resulting in incomplete emulsification. Therefore, the swept volume is hardly increased and the remaining oil after water flooding cannot be reactivated. Under similar IFT conditions, the A system has a higher sweep efficiency because the weak alkali reacts with the polar substances in the crude oil to generate certain surfactants. This leads to more complex physical and chemical changes during the displacement process, and the state of the residual oil is changed. It promotes the oil sections that are difficult to flow after water flooding to be emulsified and then extracted, so the sweep efficiency of the A system is higher than the S system. The mechanism of P flooding is completely different from that of A and S flooding. During the displacement process, water-soluble phosphorus mainly enhances the rheological properties of the displacement fluid, increases the flow resistance in the water flooding channel, delays the breakthrough time of the wetting phase, and improves the mobility. As a result, the microscopic sweep efficiency of the P system has been greatly improved, and it has been successfully increased to 61.9% based on water flooding (45.6%) [41]. The SP system has a better oil displacement effect than all the single systems (A/S/P) because of the low IFT of S and the viscosity-increasing effect of P. In the ASP system, not only is the sweep efficiency as high as 87.1%, but the oil displacement efficiency within the sweep range also reaches 94.1%. This is due to the appropriate bulk viscosity of the composite flooding system that can effectively block the water flow channel. The blocking effect allows the displacement fluid to enter the unswept oil-rich area, thereby improving the sweep efficiency. In addition, the ASP system has ultra-low IFT and strong emulsification properties, which enables more continuous clusters of residual oil to be activated and driven away. In short, the ASP system has the interfacial reaction of A, the low IFT of S, and the viscosity-increasing effect of P, so the sweeping effect and oil washing effect of the ASP system are better than that of the SP system.

A general conclusion can be drawn from the oil washing effect within the affected area in Figure 5b. In general, the oil displacement efficiency of all systems has increased slightly based on water flooding. Here, the core reason for the improvement in oil displacement efficiency lies in the expansion of the swept volume.

**Figure 6:**
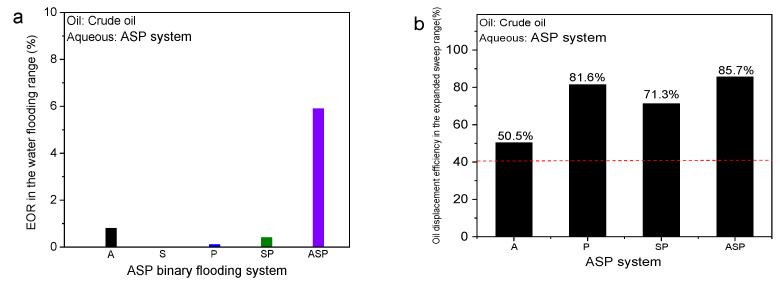
(**a**) Enhanced oil displacement efficiency in the range of water flooding. (**b**) Oil displacement efficiency in the expanded area outside the water flooding range after A/S/P/SP/ASP system flooding. The red dotted line in (**b**) represents the average oil displacement efficiency of water flooding.

Next, the EOR within the water flooding range and the expanded area outside the water flooding was calculated to determine the contribution of different systems to the oil displacement effect in Figure 6. Within the scope of water flooding, S flooding cannot activate the remaining oil after water flooding due to the existence of advantageous channels. Although P flooding increases the displacement pressure and expands the swept volume through the thickening effect, the improvement in EOR is limited. The SP system has almost no ability to start discontinuous oil ganglia bound by capillary forces after water flooding. The A system has a higher EOR than the P and SP systems because of the interface reaction. In other words, A is very essential for the initiation of residual oil after water flooding. Hence, the ASP system is effective for the residual oil at this location because of the presence of the alkali (Figure 6a). Nevertheless, the oil displacement efficiency of the ASP system reaches 85.7% in the expanded area outside water flooding (place filled with crude oil) compared with water flooding (average EOR is 40.9%). Notably, the ASP system has the best oil recovery effect in this area (Figure 6b). It is not difficult to see that the role of the interfacial reaction of A in this region is no longer obvious, but is mainly reflected in the low IFT of S and the rheological properties of P. Here, the data of the S system is not listed because the S system did not expand the affected system based on water flooding and this part of the data cannot be obtained. Next, we will elucidate the mechanism of the ASP system to significantly increase oil recovery from the dynamic changes of the residual oil in two local locations.

### 3.5. Oil Displacement Mechanism of ASP Ternary System

**Figure 7:**
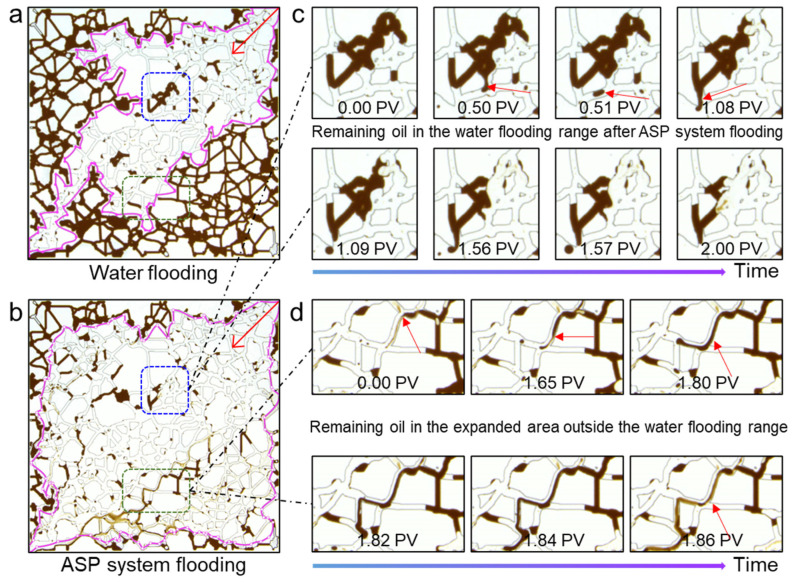
The microscopic oil displacement mechanism of the ASP ternary system. The remaining oil state after formation water (**a**) and ASP system flooding (**b**). The ASP system displaced the remaining oil in the water flooding range (**c**) and expanded the area outside the water flooding range (**d**). The pink area represents the sweep range, and the red arrows represent the displacement direction.

Figure 7 provides a detailed analysis of the complex mechanism of the ASP system to comprehensively improve oil recovery from the water flooding range and the expanded area outside the water flooding. The residual clusters after water flooding are taken as the observation object (blue area). After the ASP solution is injected, the originally immobile residual oil gradually emulsifies. Subsequently, the residual oil is entrained and flowed away in the form of small oil-in-water droplets. Under the shearing action of the porous media, the remaining oil within the water flooding range gradually decreases [42]. Observing the residual oil in the expanded area outside the water flooding (green area), it can be seen that the ASP system not only emulsifies the residual oil but also stretches it into filaments. The oil filaments are then broken and agglomerated to form small oil droplets that are transported out, which effectively breaks up the large continuous clusters of residual oil. As a whole, the ASP system can not only activate the remaining oil that remains immobile after water flooding, but also emulsify, strip, and entrain the remaining oil in the expanded area outside of water flooding. The two aspects work together to reduce the amount of remaining oil and increase the oil recovery.

## 4. Conclusions

In this paper, a weak alkali–surfactant–polymer (ASP) ternary composite flooding system formula was constructed for Daqing crude oil. Microscopic visualization equipment was used to conduct five groups (A/S/P/SP/ASP systems) of chemical slug displacement experiments after water flooding. In addition to characterizing oil displacement performance, the interfacial properties such as oil/water IFT, emulsification performance, and bulk viscosity were also tested. The microscopic oil displacement mechanism of A/S/P for synergistically improving oil recovery was comprehensively elucidated. The main experimental results are summarized as follows:

(1) In the ASP system, the active substances generated by the alkali synergize with the surfactant molecules to form a more compact interfacial film. Meanwhile, the polymer and surfactant can form a mixed adsorption layer with a higher surface charge. This enables the ASP system to obtain ultra-low interfacial tension;
(2) Alkali not only plays the role of salt to reduce the viscosity of the system but also tends to break the polymer chain. Therefore, the bulk viscosity of the ASP system is larger than that of the A and S systems but smaller than that of the P and SP systems;
(3) The low interfacial tension of the ASP system facilitates the emulsification of crude oil. Alkali decreases the viscosity of the ASP system but weakens the stability of the emulsion. The ASP system has a strong emulsifying ability, fast emulsification rate, uniform emulsion, and the ability to emulsify stripping crude oil;
(4) The ASP system simultaneously has the advantages of interfacial reaction of alkali, low interfacial tension of surfactant, and viscosity-increasing effect of the polymer, which significantly improves the sweep effect and oil washing effect. The ASP system has the highest EOR (38.0%) compared with other systems;
(5) The interfacial reaction of the alkali facilitates the mobilization of residual oil after water flooding, and the low tension of the surfactant and the rheology of the polymer can emulsify, strip, and entrain the remaining oil in the expanded area outside of water flooding, which improves the crude oil recovery of the ASP system;
(6) Ternary flooding can be used in combination with CO_2_/water alternating flooding, thus reducing the use and emission of CO_2_.

Herein, a novel ternary composite ASP flooding formula with low cost and technical feasibility is constructed, and the microscopic oil displacement mechanism is also explored, which is of great significance for the application of the ASP flooding system in Daqing Oilfield.

## Figures and Tables

**Table 1:** Physical and chemical parameters of the polymer (HPAM).

Sample	Molecular Weight (×10^4^)	Degree of Hydrolysis (mol %)	Solid Content (wt %)
Polymer	1510.0	24.1	90.7

**Table 2:** Formulation and composition of Daqing simulated formation water.

TDS (mg/L)	Na^+^ (mg/L)	Ca^2+^ (mg/L)	Mg^2+^ (mg/L)	Cl^−^ (mg/L)	SO_4_^2−^ (mg/L)	CO_3_^2−^ (mg/L)	HCO_3_^−^ (mg/L)
5683.26	1741.54	60.12	9.12	801.96	54.03	371.64	2644.85

**Table 3:** Enhanced oil recovery data of the A/S/P/SP/ASP flooding systems.

Oil Displacement System	IFT (mN·m^−1^)	Bulk Viscosity (cP)	Water Flooding (%)	A/S/P/SP/ASP Flooding (%)	EOR (%)
1.2% A	0.032	1.0	39.3%	45.6%	6.3%
0.3% S	0.020	1.0	40.5%	41.4%	0.9%
0.2% P	>10	68.1	41.6%	54.8%	13.2%
0.3% S + 0.2% P	0.044	66.2	40.0%	61.0%	20.9%
1.2% A + 0.3% S + 0.2% P	0.0085	27.7	41.7%	79.7%	38.0%

## Data Availability

The original contributions presented in the study are included in the article, further inquiries can be directed to the corresponding authors.

## Abbreviations

A: alkali system; S, surfactant system; P, polymer system; SP, surfactant–polymer binary compound system; ASP, alkali–surfactant–polymer ternary compound system.
